# An experimentally-achieved information-driven Brownian motor shows maximum power at the relaxation time

**DOI:** 10.1038/s41598-018-30495-6

**Published:** 2018-08-14

**Authors:** Dong Yun Lee, Jaegon Um, Govind Paneru, Hyuk Kyu Pak

**Affiliations:** 1 Center for Soft and Living Matter, Institute for Basic Science (IBS), Ulsan, 44919 Republic of Korea; 2 CCSS, CTP and Department of Physics and Astronomy, Seoul National University, Seoul, 08826 Republic of Korea; 3 Department of Physics, Ulsan National Institute of Science and Technology (UNIST), Ulsan, 44919 Republic of Korea

## Abstract

We present an experimental realization of an information-driven Brownian motor by periodically cooling a Brownian particle trapped in a harmonic potential connected to a single heat bath, where cooling is carried out by the information process consisting of measurement and feedback control. We show that the random motion of the particle is rectified by symmetry-broken feedback cooling where the particle is cooled only when it resides on the specific side of the potential center at the instant of measurement. Studying how the motor thermodynamics depends on cycle period *τ* relative to the relaxation time *τ*_*B*_ of the Brownian particle, we find that the ratcheting of thermal noise produces the maximum work extraction when *τ* ≥ 5*τ*_*B*_, while the extracted power is maximum near *τ* = *τ*_*B*_, implying the optimal operating time for the ratcheting process. In addition, we find that the average transport velocity is monotonically decreased as *τ* increases and present the upper bound for the velocity.

## Introduction

Brownian motors^1–3^ refer to systems that are capable of transporting Brownian particles in one direction by rectifying random thermal motion, generally operated by energy input and symmetry breaking. The design of an efficient Brownian motor operating away from equilibrium would serve as model for biological and artificial submicron scale machines. Recent advances in the field of information thermodynamics^4–20^ have made possible to realize information-driven Brownian motors that can greatly enhance the directed motion of the particles and extract useful work by rectification of thermal noise. There were several studies about a colloidal particle trapped in a potential with feedback loop^9,13,18,21,22^. One example is the theoretical study of the feedback mechanism of trap center and stiffness depending on measurement outcome^9^. On experimental side, Toyabe *et al*.^18^ showed that a colloidal particle on a spiral-staircase-like potential works as the information engine. Recently, Paneru *et al*.^22^ demonstrated a nearly perfect Brownian information engine that converts all available information into mechanical work via an error-free feedback control. However, these studies primarily focused on the extraction of useful work from information and the validation of generalized second law of thermodynamics. In this work, we realize an information-driven Brownian motor to study the motor thermodynamics depending on cycle period *τ* relative to the characteristic time *τ*_*B*_ of the Brownian particle. The motor is operated by the information obtained from the periodic measurements and asymmetric cooling of a Brownian particle trapped in a harmonic potential in contrast to the conventional Brownian motors which are operated by the periodic energy input (heating) and the asymmetrically shaped potential. We drive our feedback schemes as function of *τ* ranging from 0.1*τ*_*B*_, corresponding to a highly nonequilibrium process, to 10*τ*_*B*_, at which the system is fully relaxed, in order to find the optimal cycle period for maximum work, power extraction, and transport velocity, for what is considered to be the first time. All the prior experimentally realized feedback controlled Brownian motors have cycle period larger than their characteristic relaxation period^17,18^.

## Feedback Control Design

**Figure 1:**
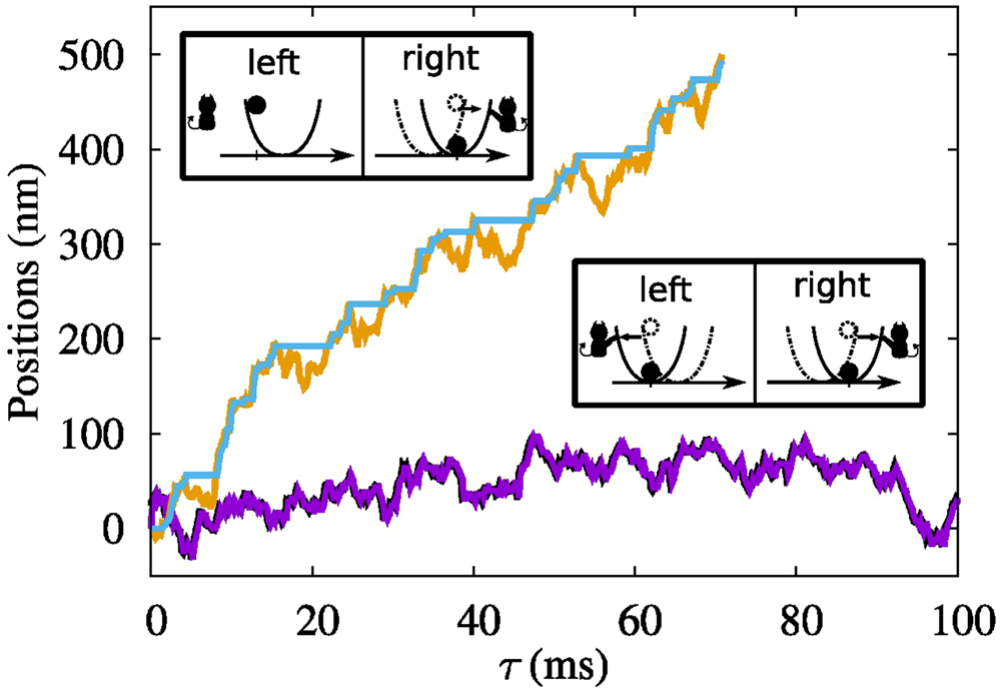
The upper and lower curves denote the trajectories for the cases of asymmetric and symmetric cooling, respectively. In the symmetric case, at each cycle, the position of particle is measured, and the center of potential is shifted to the measured position immediately after the measurement as depicted in the lower inset. The resulting trajectories for position *x* (black curve) and potential center *X* (magenta curve) show the random motions. For the asymmetric cooling, where only a particle measured at the right side of potential is cooled by shifting the potential center as seen in the upper inset, the trajectories are obviously driven in the right direction. In contrast to the symmetric case, where *X* is following *x* at every step, the potential center moves only when *x* (orange curve) is greater than *X* (blue curve) at the moment of measurement. The trajectories for both cases are measured with the period *τ* = 200 μs.

Two kinds of feedback controlled schemes are realized: the *symmetric feedback control* which behaves as an efficient cooling device and the *asymmetric feedback control* which behaves as a rectifier of thermal noise. The basic idea of our feedback schemes is illustrated in Fig. 1. A colloidal particle is confined in one dimensional harmonic potential of constant stiffness *k* = 4.6 pN/μm generated by a sharply focused laser beam and subject to periodic feedback control. Each feedback cycle consists of three processes: measurement of the particle position, control of the potential center depending on the measurement outcome, and relaxation in the fixed potential for time *τ*. The motion of the particle during the relaxation can be described by the overdamped Langevin equation^21,23^.

The lower inset of Fig. 1 illustrates the *i*th feedback cycle for the case of symmetric feedback scheme, where the particle position *x* is measured with 1 nm resolution at time *t*_*i*_ and the measurement outcome *m*_*i*_ = *x*_*i*_ is obtained. At this stage, the particle has the potential energy of *k*(*m*_*i*_ − *X*_*i*−1_)^2^/2, where *X*_*i*−1_ is the position of the potential center at the (*i* − 1)th cycle. The potential center is then shifted instantaneously (within 20 μs) to the measured position *X*_*i*_ = *m*_*i*_, cooling the particle by decreasing its potential energy to zero. Since the potential center is shifted almost instantaneously, we assume no heat exchange between the system and the heat bath during the cooling procedure. As a result, positive work is extracted from the system with the amount equal to the decrease in potential energy, *W*_*i*_ = *k*(*m*_*i*_ − *X*_*i*−1_)^2^/2. The *i*th cycle ends with the relaxation of particle for time *τ* in the updated potential during which heat energy flows from the heat bath to the system. The lower curve in Fig. 1 shows the recorded trajectory of the particle position for an interval of 100 ms when the cycle period *τ* = 200 μs. Since the potential center is shifted following the random motion of particle, the recorded trajectory shows undirected thermal diffusion.

The asymmetric feedback scheme is illustrated in the upper inset of Fig. 1. The difference from the former scheme is that the potential center is instantaneously shifted to *m*_*i*_ only when *m*_*i*_ > *X*_*i*−1_. Whereas, for *m*_*i*_ ≤ *X*_*i*−1_, no action is taken. We then wait for the same time *τ* and next cycle is repeated. The extracted work during the *i*th feedback is then given by, *W*_*i*_ = *k*/2(*m*_*i*_ − *X*_*i*−1_)^2^ if *m*_*i*_ > *X*_*i*−1_, and 0 if *m*_*i*_ ≤ *X*_*i*−1_, leading to the less work extraction on average than the symmetric case. Nevertheless, this scheme is important because it leads to one-way transportation of the particle by rectifying the thermal fluctuations which is achieved by shifting the potential center (or cooling the particle) only when *m*_*i*_ > *X*_*i*−1_. During the relaxation part of feedback cycle, the particle in the shifted potential diffuses equally in both directions, while for the case of unchanged potential, the particle diffuses more to the right due to the presence of harmonic potential wall at left; consequently, there is net flow of the particle to the right as seen in the upper curve of Fig. 1.

Since the potential center is fixed during the relaxation, the frame of relative position *x*′ = *x* − *X* can be used to describe thermodynamic quantities such as work and transport velocity. In this frame, we define two probability densities: ${p}_{i}^{s(a)}(x^{\prime} ;0)$ immediately after the *i*th feedback and ${p}_{i}^{s(a)}(x^{\prime} ;\tau )$ at the end of the *i*th relaxation of time *τ* for the case of symmetric (asymmetric) feedback scheme. We define heat dissipation from the system to the reservoir during *i*th relaxation as

1 $$\langle {Q}_{i}^{s(a)}\rangle =-\,\frac{k}{2}{\int }_{-\infty }^{\infty }dx^{\prime} {x^{\prime} }^{2}[{p}_{i}^{s(a)}(x^{\prime} ;\tau )-{p}_{i}^{s(a)}(x^{\prime} ;0)].$$

For symmetric feedback scheme, the cooling is similar to the resetting process^24^, which means that particle is localized at the center of potential immediately after the feedback, i.e., ${p}_{i}^{s}(x^{\prime} ;0)=\delta (x^{\prime} ).$ After relaxation, ${p}_{i}^{s}(x^{\prime} ;\tau )$ is described by the normal distribution whose variance is given by ${{\rm{\Delta }}}_{\tau }\equiv ({k}_{B}T/k)[1-\exp (-\tau /{\tau }_{B})]$^25^. Here, *τ*_*B*_ ≡ *γ*/2*k* is the characteristic relaxation time with dissipation coefficient *γ*, and *τ*_*B*_ = 1.86 ms in this experiment. Since ${p}_{i}^{s}(x^{\prime} ;\tau )$ is independent of the cycle index *i*, the heat dissipation per cycle is given by

2 $$\langle {Q}^{s}\rangle =-\,\frac{k}{2}{\int }_{-\infty }^{\infty }dx^{\prime} {x^{\prime} }^{2}{p}_{i}^{s}(x^{\prime} ;\tau )=-\,\frac{{k}_{B}T}{2}[1-\exp \,(\,-\tau /{\tau }_{B})],$$

where we use that the internal energy after feedback is zero. The extracted work 〈*W*^*s*^〉 by a subsequent measurement and feedback control is just given by 〈*W*^*s*^〉 = −〈*Q*^*s*^〉 due to energy conservation.

Unlike the symmetric feedback scheme, work and heat for the asymmetric feedback scheme are not given by a simple form like Eq. (1) because ${p}_{i}^{a}(x^{\prime} ;0)$ consists of not only the delta function but also contribution from the case of keeping the potential unchanged. For a large number of feedback cycles, ${p}_{i}^{a}(x^{\prime} ;\tau )({p}_{i}^{a}(x^{\prime} ;0))$ converges to the steady distribution *p*^*a*^(*x*′;*τ*) (*p*^*a*^(*x*′;0)) then *p*^*a*^(*x*′;0) is given by ${p}^{a}(x^{\prime} ;0)={p}^{a}(x^{\prime} ;\tau ){\rm{\Theta }}(-x^{\prime} )+\delta (x^{\prime} )$ ${\int }_{0}^{\infty }dy{p}^{a}(y;\tau ).$ Here, Θ(−*x*′) is the Heaviside step function, Θ(−*x*′) = 1 for *x*′ < 0, otherwise zero. The average work extraction (or heat dissipation) per cycle in the steady state can be written as

3 $$\langle {W}^{a}\rangle =-\,\langle {Q}^{a}\rangle =\frac{k}{2}{\int }_{-\infty }^{\infty }dx^{\prime} {x^{\prime} }^{2}[{p}^{a}(x^{\prime} ;\tau )-{p}^{a}(x^{\prime} ;0)]=\frac{k}{2}{\int }_{0}^{\infty }dx^{\prime} {x^{\prime} }^{2}{p}^{a}(x^{\prime} ;\tau ).$$

## Results for Symmetric Feedback Scheme

**Figure 2:**
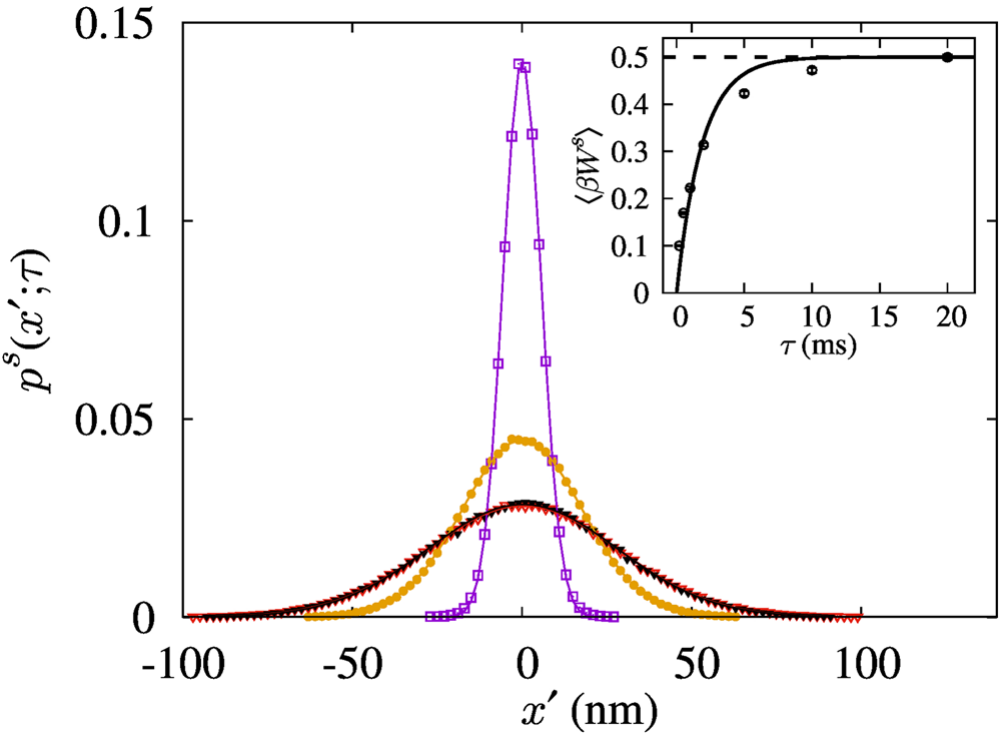
Normalized probability distributions of the particle position for various *τ* in the symmetric feedback scheme. The magenta open squares, orange solid circles and red open triangles correspond to *τ* equal to 200 μs, 2 ms, and 20 ms, respectively. The black solid triangle data correspond to the equilibrium distribution that was obtained by measuring the particle position without feedback. The solid curves are obtained by fitting the data to the normal distribution with the variance given by 2〈*W*^*s*^(*τ*)〉/*k*. Inset: Plot of average work extraction per period as a function of *τ*. The error bars denote the standard error of the mean. The dashed horizontal line corresponds to the equilibrium potential energy *k*_*B*_*T*/2. The solid curve follows $({k}_{B}T/2)\,[1-\exp (\,-\,\tau /{\tau }_{B})]$ with *τ*_*B*_ = 1.86 ms.

Figure 2 shows the normalized probability distributions of the particle position as functions of *τ* obtained experimentally for the symmetric feedback scheme, fit to the normal distribution. For *τ* < *τ*_*B*_, the particle does not have sufficient time to relax fully in the updated potential; as a result, the variance of the distribution decreases^26^. In this experiment, the smallest variance corresponds to 39 K in the temperature unit for *τ* = 200 μs. On the other hand, for *τ* ≥ 5*τ*_*B*_, the system goes to thermal equilibrium; consequently, *p*^*s*^(*x*′; *τ*) is close to the equilibrium distribution with variance of *k*_*B*_*T*/*k*. The inset of Fig. 2 shows the plot of experimentally measured average work extraction 〈*W*^*s*^(*τ*)〉 as a function of *τ*. Since 〈*W*^*s*^(*τ*)〉 is same as the average potential energy at the moment of measurement or the average heat flow from the heat bath to the system during the relaxation, it is proportional to the variance of the normal distribution. As a result, it matches well with the solid curve of $({k}_{B}T/2)\,[1-\exp \,(\,-\,\tau /{\tau }_{B})]$ and saturates to the maximum average potential energy of *k*_*B*_*T*/2 when *τ* ≥ 5*τ*_*B*_.

## Results for Asymmetric Feedback Scheme

**Figure 3:**
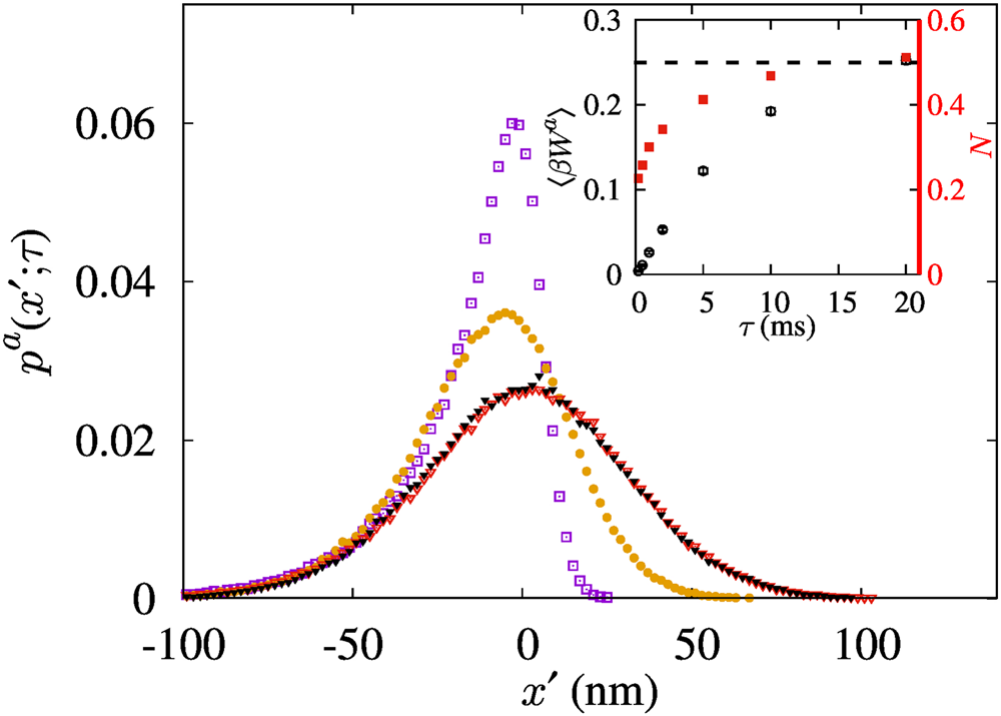
Normalized probability distributions of the particle position for various *τ* in the asymmetric feedback scheme. The purple open squares, orange solid circles and red open triangles correspond to *τ* equal to 200 μs, 2 ms, and 20 ms, respectively. The black solid triangle data correspond to the equilibrium distribution that was obtained by measuring the particle position without feedback. Inset: Plot of average work extraction per period denoted by black open circles and the probability of finding a particle at *x*′ > 0 denoted by red solid squares, as functions of *τ*. The error bars denote the standard error of the mean. The dashed horizontal line equal to *k*_*B*_*T*/4 or 1/2.

Next, we discuss the asymmetrically controlled motor depicted in Fig. 1. The probability of finding the particle to be cooled on the right side at the time of measurement is defined as $N(\tau )\equiv {\int }_{0}^{\infty }dx^{\prime} {p}^{a}(x^{\prime} ;\tau )$. After the asymmetric feedback, which is equivalent to the asymmetric resetting of *p*^*a*^(*x*′; 0) = 0 at *x*′ > 0, the probability distribution will be recovered to *p*^*a*^(*x*′; *τ*) by the following relaxation. Figure 3 displays the experimental plot of *p*^*a*^(*x*′; *τ*) as a function of *τ*. For small *τ*, where short relaxation is allowed, *p*^*a*^(*x*′; *τ*) shows a highly asymmetric form with vanishing *N*(*τ*). Since the typical length scale of diffusion is proportional to $\sqrt{\tau }$ as *τ* → 0, $N \sim \sqrt{\tau }$ in the limit of small *τ*, implying *N*(*τ*) → 0 as *τ* → 0. For *τ* ≥ 5*τ*_*B*_, the system relaxes fully; as a result, *p*^*a*^(*x*′; *τ*) becomes close to the equilibrium distribution. *N*(*τ*) also saturates to one half (see the red solid squares in the inset of Fig. 3). Since the work extraction is equal to the potential energy of the particle in *x*′ > 0 at the instant of measurement, its average 〈*W*^*a*^(*τ*)〉 per cycle is positively related to *N*(*τ*). Therefore, it increases with *τ*, and saturates to one half of the maximum average potential energy *k*_*B*_*T*/4 for *τ* = 20 ms (see the black open circles in the inset of Fig. 3). This is because the work is extracted from the fully equilibrated system with probability *N* = 1/2.

**Figure 4:**
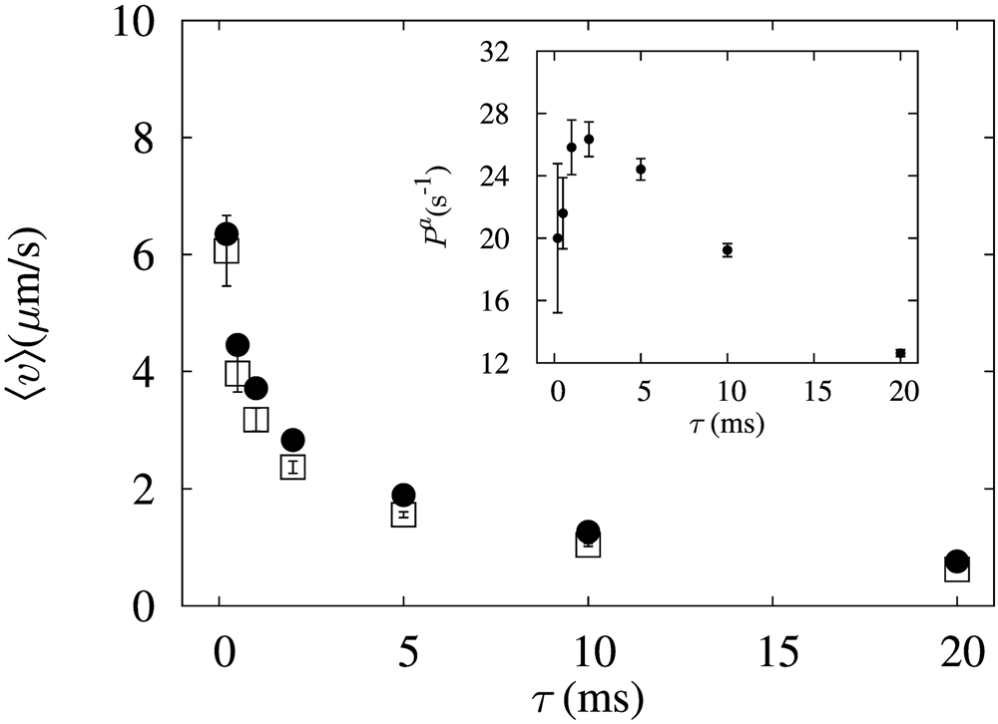
Plot of average transport velocity as a function of cycle period *τ*. Open squares correspond to the velocity induced by the asymmetric cooling and closed circles correspond to the upper bound for the velocity obtained Eq. 5. The error bars denote the standard error of the mean. Inset: power gain as function of *τ*. The error bars denote the standard error of the mean.

Inset of Fig. 4 shows the average power defined as *P*^*a*^(*τ*) = 〈*W*^*a*^(*τ*)〉/*τ*, as a function of *τ* for the case of asymmetric cooling. *P*^*a*^ is maximum near *τ* = *τ*_*B*_ and vanishes for both *τ* → 0 and *τ* → ∞. In the steady state, 〈*W*^*a*^(*τ*)〉 is equivalent to the increment of average internal energy by the heat flow from the heat bath per cycle. For *τ* → 0, where the diffusion length is vanishingly small, the average work extraction per cycle can be approximated as 〈*W*^*a*^(*τ* → 0)〉 ~ *Nτ*. Thus, ${P}^{a}(\tau \to 0) \sim \sqrt{\tau }$ implying that power vanishes at *τ* → 0. As mentioned above, since 〈*W*^*a*^(*τ*)〉 is equivalent to the average heat flow from the heat bath, it is a relaxation quantity which increases with *τ* fast at *τ* < *τ*_*B*_, and slowly around *τ* > *τ*_*B*_, eventually saturating to *k*_*B*_*T*/4 in long time limit. This explains qualitatively why the characteristic time *τ*_*B*_ is the natural time scale at which the increasing power is turned into a decreasing function, forming the maximum near *τ*_*B*_, in the end decaying as *P*^*a*^ ~ *τ*^−1^ in time. This prediction of power law behavior in both limits agrees qualitatively with the experimental data. Our motor is optimized as a function of cycle period only; however, it is worth to note that the performance of the engine can depend upon other control parameters too^9,20^. Therefore, the current protocol is not optimal for maximum power extraction.

For most practical application, it is important to know how fast the motor can transport the particle. We measured the average transport velocity 〈v〉 per cycle given by,

4 $$\langle {\rm{v}}\rangle =\frac{1}{\tau }{\int }_{-\infty }^{\infty }dx^{\prime} [{p}^{a}(x^{\prime} ;\tau )-{p}^{a}(x^{\prime} ;0)]x^{\prime} =\frac{1}{\tau }{\int }_{0}^{\infty }dx^{\prime} {p}^{a}(x^{\prime} ;\tau )x^{\prime} .$$

Validity of the predicted exponents for power and the velocity can be also checked by using the following inequality:

5 $${N}^{-1}{\int }_{0}^{\infty }dx^{\prime} {p}^{a}(x^{\prime} ;\tau ){[x^{\prime} -\overline{x^{\prime} }]}^{2}\ge 0,$$

where $\overline{x^{\prime} }={N}^{-1}{\int }_{0}^{\infty }dx^{\prime} {p}^{a}(x^{\prime} ;\tau )x^{\prime} .$ Using Eqs (3) and (5) in Eq. (4), we derive the following bound for average transport velocity,

6 $$\langle {\rm{v}}\rangle \le \frac{1}{\tau }\sqrt{\frac{2N\langle {W}^{a}\rangle }{k}}.$$

This means that 〈v〉 is bounded by $\sqrt{{P}^{a}}$ and $\sqrt{N/\tau }$. As discussed before, $\sqrt{N\langle {W}^{a}\rangle } \sim \tau$ for *τ* → 0, and as *τ* is increased it grows linearly first and later slower than the linear function of *τ*, eventually saturates. Therefore, the bound is decreased as *τ* is increased, implying that the velocity also decreases and vanishes in the long time limit, which agrees well with experimental data as shown in Fig. 4.

In conclusion, the significance of this paper is to realize an information driven Brownian motor with the help of modern experimental techniques that have nanometer and microsecond resolution. By measurement and asymmetric feedback cooling, we could rectify the random thermal motion in contrast to the conventional Brownian motors which need periodic energy input (heating) and asymmetrically shaped potential. For cycle period shorter than the characteristic relaxation time, the highly asymmetric distribution enhances the transport velocity of the motor, but its power is suppressed, suggesting that the motor needs to be operated with cycle period equal to the characteristic relaxation period at which power is maximized. This study not only offers the better understanding of nonequilibrium thermodynamics of small systems, but also would assist the design of future micro or nano scale machines where fluctuations are inevitable.

## Methods

The details of our experimental setup can be found in our previously published research^22^, and hence only a brief description is given here. A laser with 1064 nm wavelength is fed to an acoustic optical deflector (AOD) (Isomet, LS110A-XY) that is controlled via an analog voltage controlled Radio Frequency (RF) synthesizer driver (Isomet, D331-BS) and used for trapping the particle. A second laser with 980 nm wavelength is used for tracking the particle position. The AOD is properly mounted at the back focal plane of the objective lens so that *k* remains constant when periodic shifting of the potential center is done (see Figs 2 and 3 where the variance of the distribution without feedback is same with that for *τ* = 20 ms). A Quadrant Photo Diode (QPD) (S5980, Hamamatsu) is used to detect the particle position. The signal from QPD is pre-amplified by a signal amplifier (On-Trak Photonics, Inc., OT-301) and sampled at every *τ* with a Field-Programmable Gate Array (FPGA) data acquisition card (National Instruments, PCI-7830R). Our system is capable of measuring the particle position with 1 nm resolution at the sampling rate of 200 μs which is at least five times better than the previously reported video microscopy based feedback controlled system^18^. The periodic feedback control is realized using Labview programmed on the FPGA target. The sample cell consists of highly dilute solution of 2.0 μm diameter polystyrene particles suspended in deionized water. The trapping laser power at the sample stage is maintained at ~3 mW. The laser power of the tracking laser is ~5% of the trapping laser power. All experiments were carried out at fixed temperature of 297.0 ± 0.1 K at which the viscosity of water is 0.911 mPa s. The trap stiffness was calibrated using two different techniques based on the equipartition theorem and Boltzmann distribution^27^. The trap stiffness is then obtained by averaging the values obtained by those two techniques and was found to be 4.60 pN/μm in this experiment.
